# The Clinical Potential of Oral Microbiota as a Screening Tool for Oral Squamous Cell Carcinomas

**DOI:** 10.3389/fcimb.2021.728933

**Published:** 2021-08-18

**Authors:** Xinxuan Zhou, Yu Hao, Xian Peng, Bolei Li, Qi Han, Biao Ren, Mingyun Li, Longjiang Li, Yi Li, Guo Cheng, Jiyao Li, Yue Ma, Xuedong Zhou, Lei Cheng

**Affiliations:** ^1^State Key Laboratory of Oral Diseases & West China Hospital of Stomatology & National Clinical Research Center for Oral Diseases, Sichuan University, Chengdu, China; ^2^Department of Operative Dentistry and Endodontics, West China Hospital of Stomatology, Sichuan University, Chengdu, China; ^3^West China School of Public Health and West China Fourth Hospital, Sichuan University, Chengdu, China; ^4^Laboratory of Molecular Translational Medicine, Centre for Translational Medicine, Key Laboratory of Birth Defects and Related Diseases of Women and Children, Ministry of Education, West China Second University Hospital, Sichuan University, Chengdu, China

**Keywords:** oral microbiota, OSCC, machine learning methods, diagnose, sequencing

## Abstract

**Introduction:**

The oral squamous cell carcinoma (OSCC) is detrimental to patients’ physical and mental health. The prognosis of OSCC depends on the early diagnosis of OSCC in large populations.

**Objectives:**

Here, the present study aimed to develop an early diagnostic model based on the relationship between OSCC and oral microbiota.

**Methods:**

Overall, 164 samples were collected from 47 OSCC patients and 48 healthy individuals as controls, including saliva, subgingival plaque, the tumor surface, the control side (healthy mucosa), and tumor tissue. Based on 16S rDNA sequencing, data from all the five sites, and salivary samples only, two machine learning models were developed to diagnose OSCC.

**Results:**

The average diagnostic accuracy rates of five sites and saliva were 98.17% and 95.70%, respectively. Cross-validations showed estimated external prediction accuracies of 96.67% and 93.58%, respectively. The false-negative rate was 0%. Besides, it was shown that OSCC could be diagnosed on any one of the five sites. In this model, *Actinobacteria*, *Fusobacterium*, *Moraxella*, *Bacillus*, and *Veillonella* species exhibited strong correlations with OSCC.

**Conclusion:**

This study provided a noninvasive and inexpensive way to diagnose malignancy based on oral microbiota without radiation. Applying machine learning methods in microbiota data to diagnose OSCC constitutes an example of a microbial assistant diagnostic model for other malignancies.

## Introduction

Oral cancer is a significant threat to patients’ physical and mental health. According to the Global Cancer Statistics (Bray et al., 2018), an estimated 350,000 new cases and 170,000 deaths from oral cavity cancers occurred in 2018. Most global oral squamous cell carcinoma (OSCC) cases are diagnosed in Asia. In developing countries, in particular, oral cancers rank the eighth most common cancers in males. Worryingly, the incidence of the oral cavity cancers appears to be increasing in many parts of the world (Simard et al., 2014). The most common oral cancer is OSCC, with a 95% rate. The prognosis for oral cancers is notably poor, with a mean all-stage, 5-year survival rate of <50% (Kujan et al., 2005).

Therefore, it is essential to diagnose OSCC at an early stage, especially in large populations, and the prognosis of the treatment could benefit from the early detection of OSCC. In the diagnosis of OSCC and many other tumors, pathologic diagnosis is the gold standard, and radiologic examinations provide useful supplementary data. However, it is difficult to apply these traditional methods as primary diagnostic methods for OSCC in large populations due to their invasive, radioactive, and expensive nature. Therefore, an effective, convenient, and noninvasive method is necessary as a screening tool for OSCC in large populations.

In recent years, many investigations have explored the association between oral bacteria and OSCC (Ahn et al., 2012; Pushalkar et al., 2012; Schmidt et al., 2014). Therefore, oral bacteria might be a potential biomarker to develop a promising early diagnostic method for OSCC. However, we still face considerable challenges in developing a novel diagnostic model based on oral bacteria. First, efforts are underway to find out the core microbiome or species for OSCC diagnosis. Previous studies have investigated the relationship between some single species and OSCC, including *Porphyromonas gingivalis* (Chang et al., 2019; Park et al., 2019; de Mendoza et al., 2020) and *Staphylococcus aureus* (Wang et al., 2019). Investigators have also indicated the differences in the oral microbiome between OSCC patients and healthy individuals *via* bioinformatics analysis. Some other previous studies have indicated significant losses in the richness and diversity of oral microbiota in OSCC patients compared with healthy subjects. The relative frequencies of *Streptococcus*, *Dialister*, and *Veillonella* species differentiate the tumor from a healthy state (Guerrero-Preston et al., 2016). Other studies (Krogh et al., 1987) found significantly higher frequencies of *Porphyromonas*, *Actinomycetes*, *Haemophilus*, and *Enterobacter* species on the surface of OSCC tissues. Hooper et al. demonstrated that microbial diversity increased in tumor tissues by using 16S rDNA sequencing technology (Hooper et al., 2007). However, the exact core microbiome remains unclear, and thus, diagnostic models were not established to detect OSCC based on the microbiome.

Second, the oral cavity is a complicated environment, and the microbiome is different in different sites, including the tongue, teeth, mucous membranes, palate, and gums (Aas et al., 2005; Avila et al., 2009; Zarco et al., 2012). Segata et al. reported that the composition of microbial communities varies in seven oral cavity surfaces, demonstrating that the buccal mucosa, keratinized gingiva, hard palate, saliva, tongue, tonsils, throat, and subgingival and supragingival plaques were distinct more or less (Segata et al., 2012). Therefore, it is necessary and vital to determine which site should be selected to analyze the microbiome for OSCC diagnosis.

This study showed that OSCC could be diagnosed based on oral microbiota, and a diagnostic model could be developed with the help of machine learning methods. Moreover, the microbiota in the saliva, subgingival plaque, tumor surface, the control side (normal mucosa), and intratumoral tissue were useful for OSCC diagnosis. What is more, this diagnostic model can effectively avoid missed diagnoses; therefore, it is a potential early OSCC diagnostic method for large populations.

## Materials and Methods

### Study Design

This study consisted of three stages. In stage I, the demographic data and microbiome were characterized using descriptive methods to provide a clear profile of both internal and external samples and the whole study data. Also, the microbiome and demographic data were analyzed using exploratory methods to test the study assumption, i.e., whether OSCC patients have microbiome patterns different from those of healthy people. In stage II, random forests were developed to determine the different patterns and further analyze the specified operational taxonomic unit (OTU) role in the differences between microbiome patterns of healthy and OSCC individuals. In stage III, *post hoc* analyses were carried out to evaluate the different aspects of the performance of the diagnostic model developed in stage II, i.e., external discrimination capacity and its reliability on the sample size of the random forest prediction model based on the oral microbiome.

### Participant Information

The institutional review board of the West China Hospital Stomatology of Sichuan University approved the study (approval number: WCHSIRB-D-2013-047). All the patients provided written informed consent forms before sample collection.

The sample collection protocol conformed to the Manual of Procedure for Human Microbiome Project Core Microbiome Sampling Protocol A HMP Protocol #07-001 (McInnes and Cutting, 2010; Segata et al., 2012; Sturød et al., 2020). There were 47 OSCC patients, all from China, who met the inclusion criteria, which required the use of no alcohol, no tobacco, no antibiotics, no cortisone, no cytokines (which could provoke the immune system like interleukin), and no immunosuppressant drugs like methotrexate six months before the sampling procedure. The age of the patients ranged from 34 to 78 years. The patients with DMF >4, calculus index ≥2, and oral fungal or mucosal diseases were excluded (Kalogirou and Sklavounou-Andrikopoulou, 2017; Xun et al., 2018). The control group followed the same criteria.

All patients were sampled before treatment to ensure that the microbiome was not affected by chemotherapy, radiotherapy, and oral prophylaxis. Of the 47 OSCC patients, 47 salivary samples (the saliva group), 18 subgingival plaque samples (the pla group), 21 surfaces of tumor samples (the tum–muc group), 16 control side of healthy mucosa samples (the con–muc group), and 16 tumor tissue samples (the tum group) were collected (**Table 1**). OTU composition is a whole community structure that reflects various conditions of the microenvironment, and it is affected by factors such as diet, nutrition, and living habits. Therefore, the OTU composition of samples from different regions might be significantly different. Therefore, if this factor is not eliminated and only local or single-source samples are selected for the construction of the model, regional differences might cover it when applied to the population in other regions, resulting in unsatisfactory prediction performance. Forty-six healthy individuals were included as a control, consisting of 21 salivary samples from the same region as patients in Sichuan Province, and 25 salivary samples from another center, Peking University Hospital of Stomatology (Xun et al., 2018), in Beijing, to avoid this error (**Table 1**).

**Table 1:** Samples in different groups.

	OSCC	Healthy control	External set	Total
	(n = 47)	(n = 21)	(n = 25)	(n = 93)
con_muc	16			16
Pla	18			18
Saliva	47	21	25	93
Tum	16			16
Tum_muc	21			21
Overall	118	21	25	164

OSCC, oral squamous cell carcinoma.

### Sample Collection

The participants were asked not to take in any food and not brush or floss for at least 12 h before the sample collection session. The protocol for sample collection in each site followed the Manual of Procedure for Human Microbiome Project: Core Microbiome Sampling Protocol A (HMP Protocol #07-001) (McInnes and Cutting, 2010; Segata et al., 2012; Sturød et al., 2020). The participants were taught to stop swallowing for 1 min and collect 5 ml of saliva in 50-ml Falcon tubes for saliva collection. For plaque collection, buccal swabs were used to take plaque samples from the participants, which were stored in 2-ml EP tubes. For the bacterial flora on the oral mucosa, swabs were used to wipe the lesion and the other side of the oral mucosa for 10 s, respectively, avoiding the tooth and internal tumor. All the samples were then transferred into phosphate-buffered saline (PBS) solution and stored at −80°C immediately. For the internal tumor, dental instruments were disinfected to cut the internal tumor into 1 × 1 × 1-cm^3^ cubes on a sterile platform; the tumor samples were then steeped in sterile povidone-iodine for 3 min and vortexed several times using 500 µl of PBS. The tumor samples were divided into two parts, with one being steeped in Tris-EDTA buffer (pH = 7.4) stored at −80°C and with the other one being used for cultivation (McInnes and Cutting, 2010).

### DNA Extraction and PCR Amplification

Microbial DNA was extracted from all the samples using the E.Z.N.A.^®^ soil DNA Kit (Omega Bio-Tek, Norcross, GA, USA) according to the manufacturer’s protocols (Zhu et al., 2015; Wang et al., 2016; Li et al., 2019). The V4–V5 region of the bacterial 16S ribosomal RNA gene was amplified by PCR (95°C for 2 min, followed by 25 cycles at 95°C for 30 s, 55°C for 30 s, and 72°C for 30 s, and a final extension at 72°C for 5 min) using primers 515F 5′-barcode-GTGCCAGCMGCCGCGG)-3′ and 907R 5′-CCGTCAATTCMTTTRAGTTT-3′ (Li et al., 2018; Xie et al., 2018; Zhou et al., 2018), where the barcode is an eight-base sequence unique to each sample. PCRs were performed in triplicate in a 20-μl mixture containing 4 μl of 5× FastPfu buffer, 2 μl of 2.5 mM of dNTPs, 0.8 μl of each primer (5 μM), 0.4 μl of FastPfu polymerase, and 10 ng of template DNA.

### Illumina MiSeq Sequencing

Amplicons were extracted from 2% agarose gels, purified using the AxyPrep DNA Gel Extraction Kit (Axygen Biosciences, Union City, CA, USA) according to the manufacturer’s instructions, and quantified using QuantiFluor™-ST (Promega, USA) (Xie et al., 2018). According to the standard protocols, purified amplicons were pooled in equimolar and paired-end sequenced (2 × 300) on an Illumina MiSeq platform. The Sequencing Depth of all samples was enough for analysis. The rarefaction analysis and read count statistics of all samples are shown in the **Supplementary Material** (**Figure S2** and **Table S4**). The raw reads were deposited in the National Center for Biotechnology Information (NCBI) Sequence Read Archive (SRA) database (Accession Number: SRP119028) (Zhu et al., 2015; Wang et al., 2016; Yin et al., 2016; Xie et al., 2018).

### Processing of Sequencing Data

Raw FASTQ files were demultiplexed and quality-filtered using QIIME (Version 1.9.1) with the following criteria (Caporaso et al., 2010): i) the 300-bp reads were truncated at any site with an average quality score of <20 over a 50-bp sliding window, discarding the truncated reads that were shorter than 50 bp. ii) Exact barcode matching, two nucleotide mismatches in primer matching, and reads containing ambiguous characters were removed. iii) Only sequences that overlapped longer than 10 bp were assembled according to their overlap sequence. Reads that could not be assembled were discarded.

OTUs were clustered with 97% similarity cutoff using UPARSE Version 7.1 (http://drive5.com/uparse/), and chimeric sequences were identified and removed using UCHIME. The taxonomy of each 16S rRNA gene sequence was analyzed by RDP Classifier (Cole et al., 2005) (http://rdp.cme.msu.edu/) against the silva (SSU123) 16S rRNA database using a confidence threshold of 70% (Dewhirst et al., 2010).

### Statistical Analysis

In stage I, the demographic and microbiome characteristics of the subjects were presented. An exploratory analysis was carried out to explore the potential capacity of pattern differences between samples from healthy and OSCC individuals. The Shannon index, Chao index, Simpson diversity index (Chao et al., 1992; Chao and Shen, 2003), beta diversity index, network analysis, and functional analysis were used to explore whether the microbiome profiles in samples differed between OSCC and healthy individuals.

In stage II, since exploratory analysis showed that microbiome patterns differed between OSCC and healthy individuals, random forests were developed to show that such a pattern of the total microbiome in healthy subjects was different from that in OSCC individuals. The proper discriminations of this algorithm in high-dimensional datasets have been shown in various fields. Based on the model, the OTUs with great importance in distinguishing OSCC patients from healthy individuals were also extracted to provide clues for further studies on the mechanism of interaction of microbiome and cancer incidence.

In stage III, further analyses were carried out on the prediction model based on the random forests to evaluate the external prediction capacity and the dependence on the sample size.

To evaluate external prediction capacity, although the algorithms based on CART, bagging, and bootstrap have strong resistance against the overfitting, still in practice in some cases, such prediction models cannot perform well in external datasets. Therefore, a batch of cross-validations was carried out. In each cross-validation, a fixed proportion of samples was first randomly selected as the training set to build random forests. The rest of the samples used to test the forests’ prediction capacity were used to predict whether the forests could correctly discriminate the OSCC patients from healthy individuals in the external population. Given that all the samples in the test set would not be used to train the forests, each time, the forests were tested using an external validation set. This process was repeated for large numbers to ensure that each sample would be in training and test sets for at least once. The average performance over the tests would be used to evaluate the expected external discrimination capacity of OSCC patients using random forests based on the microbiome.

As in cross-validation, not all the samples would be used to train the model, and the prediction capacity would decrease due to the loss of sample size. Therefore, it is of interest how many samples can build a reliable prediction model and whether the prediction capacity can be improved by introducing more samples. Therefore, different batches of cross-validations with different sample sizes of the training set were carried out to evaluate how the prediction capacity changes in terms of the sample size.

## Results

### Characteristics and Exploratory Analysis

**Figure 1:**
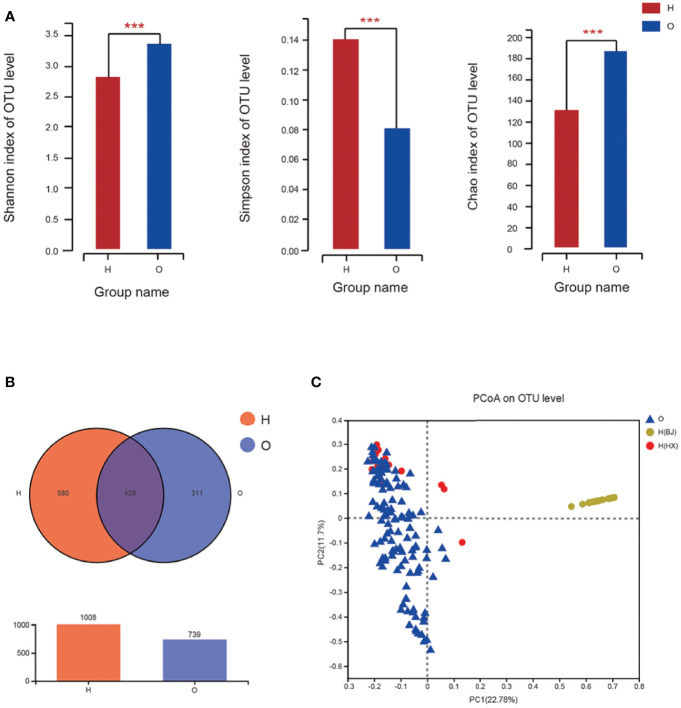
The diversities of oral microbiome in OSCC patients and healthy individuals. **(A)** Shannon, Chao and Simpson Indexes of all OTUs with relative importance greater than 0. 01 between OSCC patients and healthy control. **(B)** Venn graph between OSCC patients and healthy control. **(C)** PCoA of bray_curtis between OSCC and healthy individuals. O represented OSCC patients, H represented healthy people, and H(HX) means people from West China College of Stomatology, H(BJ) means people from Peking University Hospital of Stomatology (* means 0.01 < P ≤ 0.05, ** means 0.001 < P ≤ 0.01, *** means P ≤ 0.001).

The diversity indexes, i.e., Shannon, Chao, and Simpson indexes, showed that the diversities of oral microbiome increased significantly in OSCC patients compared with healthy individuals (**Figure 1A**).

A Venn graph (**Figure 1B**) was used to determine the number of common and distinguished OTUs between OSCC patients and healthy controls. Samples with similar levels of 97% OTU were used for the analysis. OSCC patients and the healthy group exhibited significant differences in the OTU level, with only 428 out of 1,747 OTUs in common; 311 of 1,747 OTUs were unique for OSCC patients.

The Bray–Curtis principal coordinate analysis (PCoA) showed that healthy individuals’ microbial community was concentrated, while the microbial community of patients was relatively discrete. Besides, the microbiome in samples from both OSCC and healthy individuals from the same center (West China College of Stomatology), i.e., OSCC and healthy control group, was similar. In contrast, those from the external center (Peking University Hospital of Stomatology) exhibited a different pattern (**Figure 1C**). This result supported our suspicion that microbiome profiles might differ significantly between different populations from different regions rather than those between OSCC and healthy individuals. Therefore, if the prediction model were built only with samples from a local or internal set of samples, its generalizability would be significantly limited, and the application of such a prediction model to external populations might be inappropriate. This is also evaluated by external prediction evaluation in stage III.

The key OTU phylotypes in OSCC patients and the healthy group were analyzed, which showed different phyla in the two groups. Five locations (saliva, subgingival plaque, tumor surface, normal mucosa in the control side, and intratumoral tissue) were sampled to investigate the frequencies of oral microbial communities in OSCC patients. All the results are presented in the **Supplementary Material**. This raised the interesting question of whether different sampling sites affected the model diagnosis.

These exploratory results implied that the microbiome pattern between the healthy and OSCC subjects was significantly different. The significant differences suggested that the oral microbiome does have the potential capacity to discriminate the OSCC patients from all the individuals.

### Phylogenetic Profiles of Oral Microbial Communities in Oral Squamous Cell Carcinoma Patients

**Figure 2:**
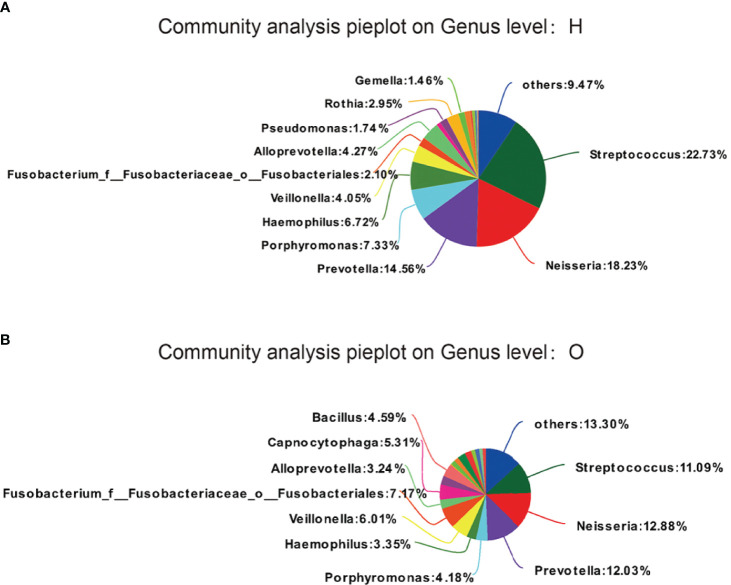
OTUs phylotypes in healthy group **(A)** and OSCC patients **(B)**, analysed by Wilcoxon rank-sum test with Benjamini-Hochberg method.

We examined the similarities and differences of genera present in the healthy group and as depicted in **Figures 2A, B**. Phylotypes with a median relative abundance larger than 0.01% of total abundance were included for comparison. To identify key OTU phylotypes in OSCC patients and healthy group, abundances of OTUs were analyzed by Wilcoxon’s rank-sum test with the Benjamini–Hochberg method.

The OTUs representing different phyla were not similar between the two groups. The healthy group was observed to contain *Streptococcus* (22.73%), followed by *Neisseria* (18.23%), *Prevotella* (14.56%), *Porphyromonas* (7.33%), *Haemophilus* (6.72%), and *Veillonella* (4.05%). The OSCC group was found to contain *Streptococcus* (11.09%), followed by *Neisseria* (12.88%), *Prevotella* (12.03%), *Porphyromonas* (4.18%), *Haemophilus* (3.35%), and *Veillonella* (6.01%).

**Figure 3:**
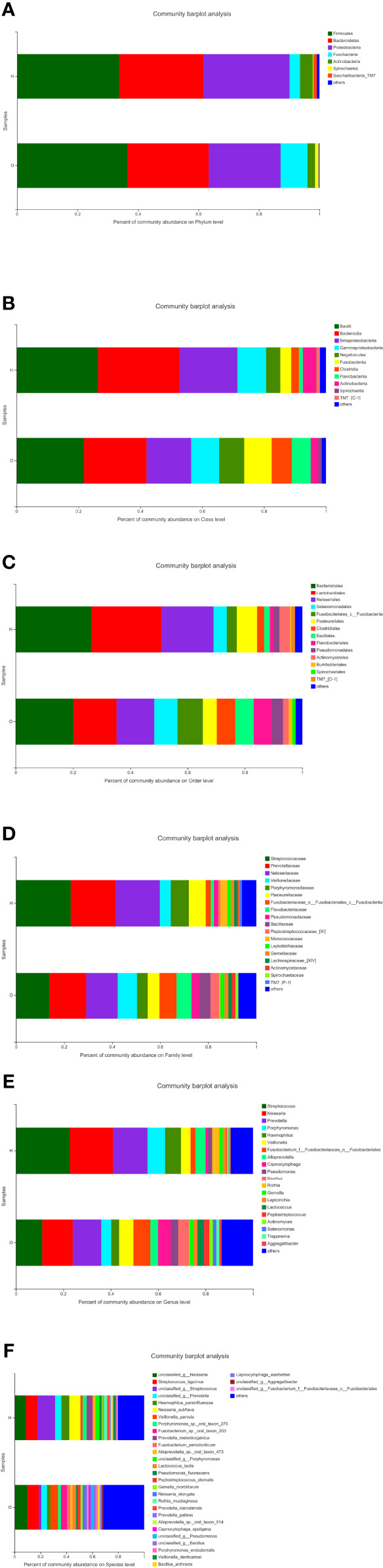
Stacked column plots representing comparison of relative abundance of bacterial taxa between healthy and oral squamous cell carcinoma (OSCC) groups at phylum **(A)**, class **(B)**, order **(C)**, family **(D)**, genus **(E)**, and species **(F)**.

The stacked column plots also showed the differences between the two groups in terms of phylum (**Figure 3A**), class (**Figure 3B**), order (**Figure 3C**), family (**Figure 3D**), genus (**Figure 3E**), and species (**Figure 3F**). Overall, the abundance of the OSCC group was higher than that of the healthy group. On the phylum level, there were less Bacteroidetes and Proteobacteria and more Firmicutes and Fusobacteria in the OSCC group. On the class level, there were less Bacilli, Bacteroidia, and Betaproteobacteria and more Negativicutes in the OSCC group. On the order level, there were less Bacteroidales, Lactobacillales, and Neisseriales and more Selenomonadales in the OSCC group. On the family level, there were less Streptococcaceae, Prevotellaceae, and Neisseriaceae and more Veillonellaceae in the OSCC group. On the genus level, there were less *Streptococcus*, *Neisseria*, and *Prevotella* and more *Veillonella* and *Fusobacterium* in the OSCC group. On the species level, there were less *Haemophilus* and more *Veillonella* in the OSCC group.

**Figure 4:**
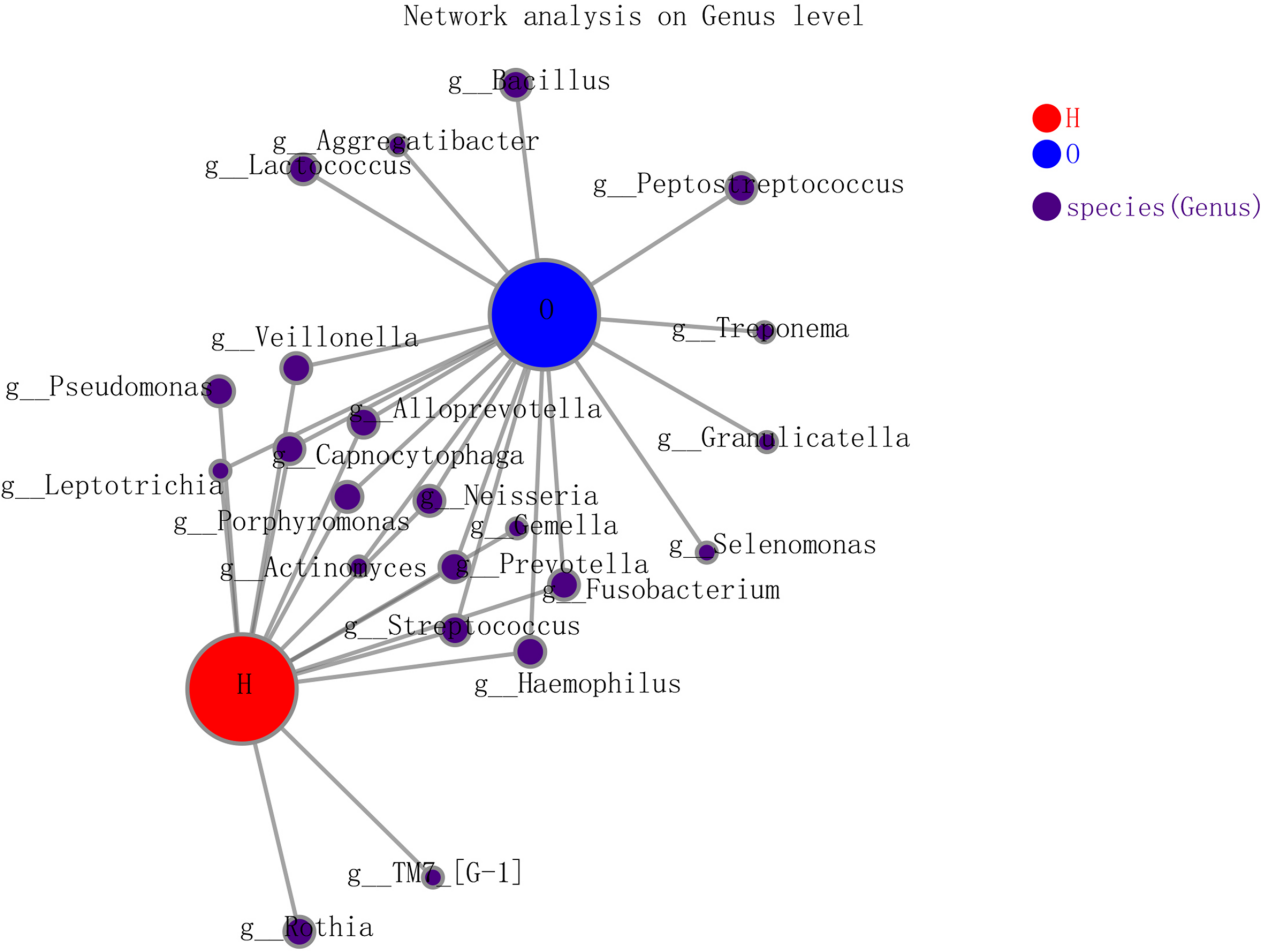
The co-occurrence network deduced from bacteria enriched in oral squamous cell carcinoma (OSCC) group and healthy group.

In the co-occurrence network deduced from bacteria enriched in the OSCC group and healthy group, the node in the network represented the sample node or the genus node, and the line between the sample node and the species node represented that the sample contains the genus. **Figure 4** shows the genus with abundance greater than 50. Both groups contained *Veillonella*, *Alloprevotella*, *Capnocytophaga*, *Neisseria*, *Gemella*, etc. Only the healthy group contained *Rothia*, and only the OSCC group contained *Lactococcus*, *Aggregatibacter*, *Peptostreptococcus*, etc.

Analysis of similarities (ANOSIM) (**Table 2**) was significant for the overall model (R^2^ = 0.13291, p = 0.05), and pairwise comparisons revealed a significant difference between control subjects who remained healthy and those with OSCC. Although the coefficient of determination is very low, there is a difference between the two groups. On the one hand, it is suggested that there can be a clear difference between the two, which can be used as an auxiliary diagnosis of OSCC; on the other hand, this mode difference may not be large and may lack discrimination in particular cases. Meanwhile, considering the number of OTUs, the samples size is relatively small, so a special method is needed to identify such slight differences. This is the machine learning diagnostic model mentioned later.

**Table 2:** ANOSIM in healthy group and OSCC patients.

	Df	SumsOfSqs	MeanSqs	F.Model	R^2^	Pr(>F)
Group	1	6.864	6.8643	24.832	0.13291	0.001
Residuals	162	44.782	0.2764	0.86709		
Total	163	51.646	1			

ANOSIM, analysis of similarities; OSCC, oral squamous cell carcinoma.

### Abundance of Oral Microbial Communities in Oral Squamous Cell Carcinoma

To illustrate that the location in the oral cavity has an effect on the microbiota of the particular niche (saliva, subgingival plaque, surface of tumor, normal mucosa in the control side, and intratumoral tissue), we sampled microbiota in these five locations.

**Figure 5:**
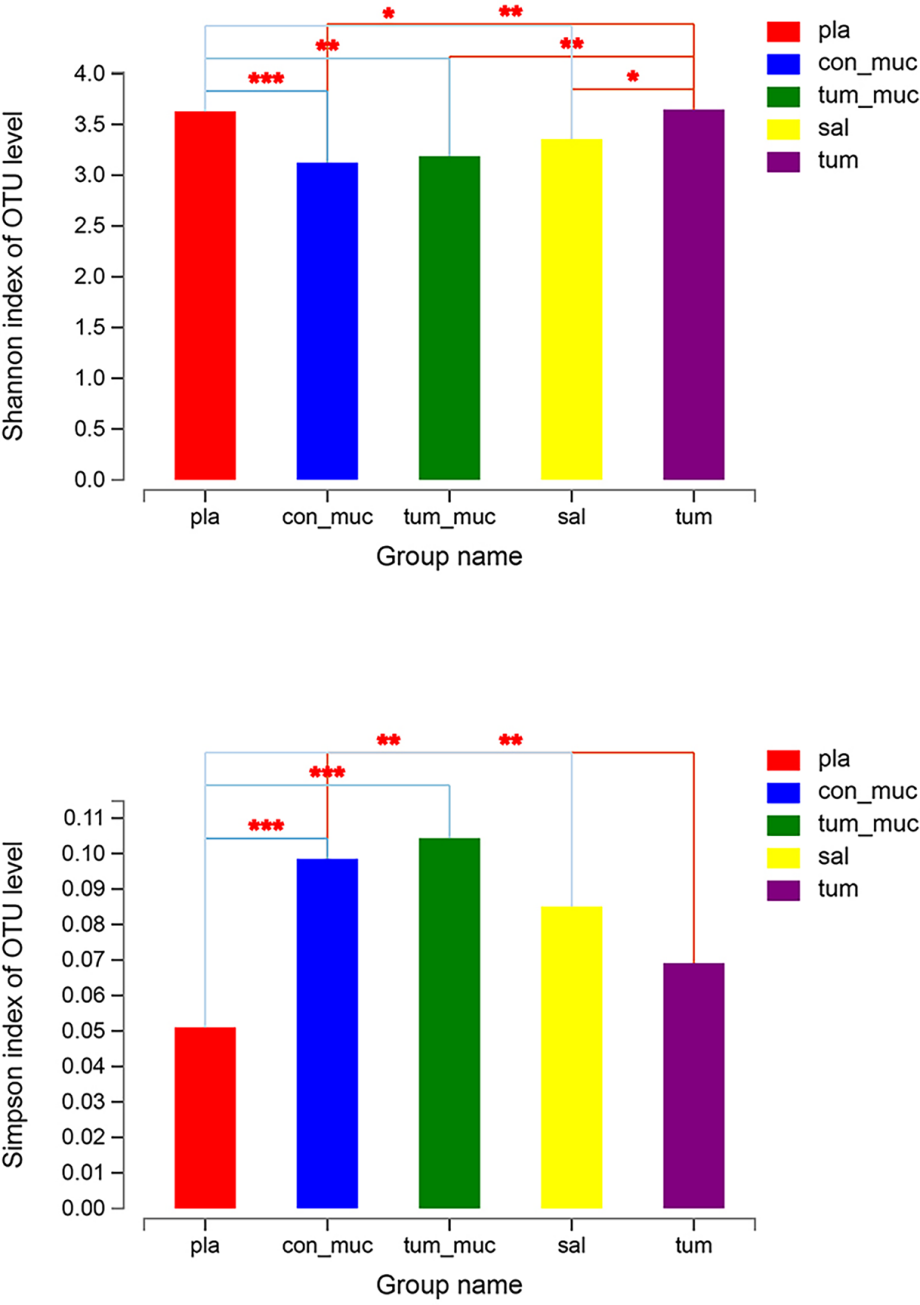
Simpson index and Shannon index of microorganisms in subgingival plaque, normal mucosa, surface of tumor, saliva and intratumor tissue (* means 0.01 < P ≤ 0.05, ** means 0.001 < P ≤ 0.01, *** means P ≤ 0.001).

The Simpson index and Shannon index reflected the diversity of microorganisms in saliva, subgingival plaque, surface of tumor, normal mucosa, and intratumoral tissue. According to **Figure 5**, there were significant differences in the Simpson index and Shannon index of these five locations, which indicated that the diversity in different parts of the oral cavity was different. Among them, the Simpson index and Shannon index in intratumoral tissue were the highest, indicating that the diversity in intratumoral tissue was relatively high.

**Figure 6:**
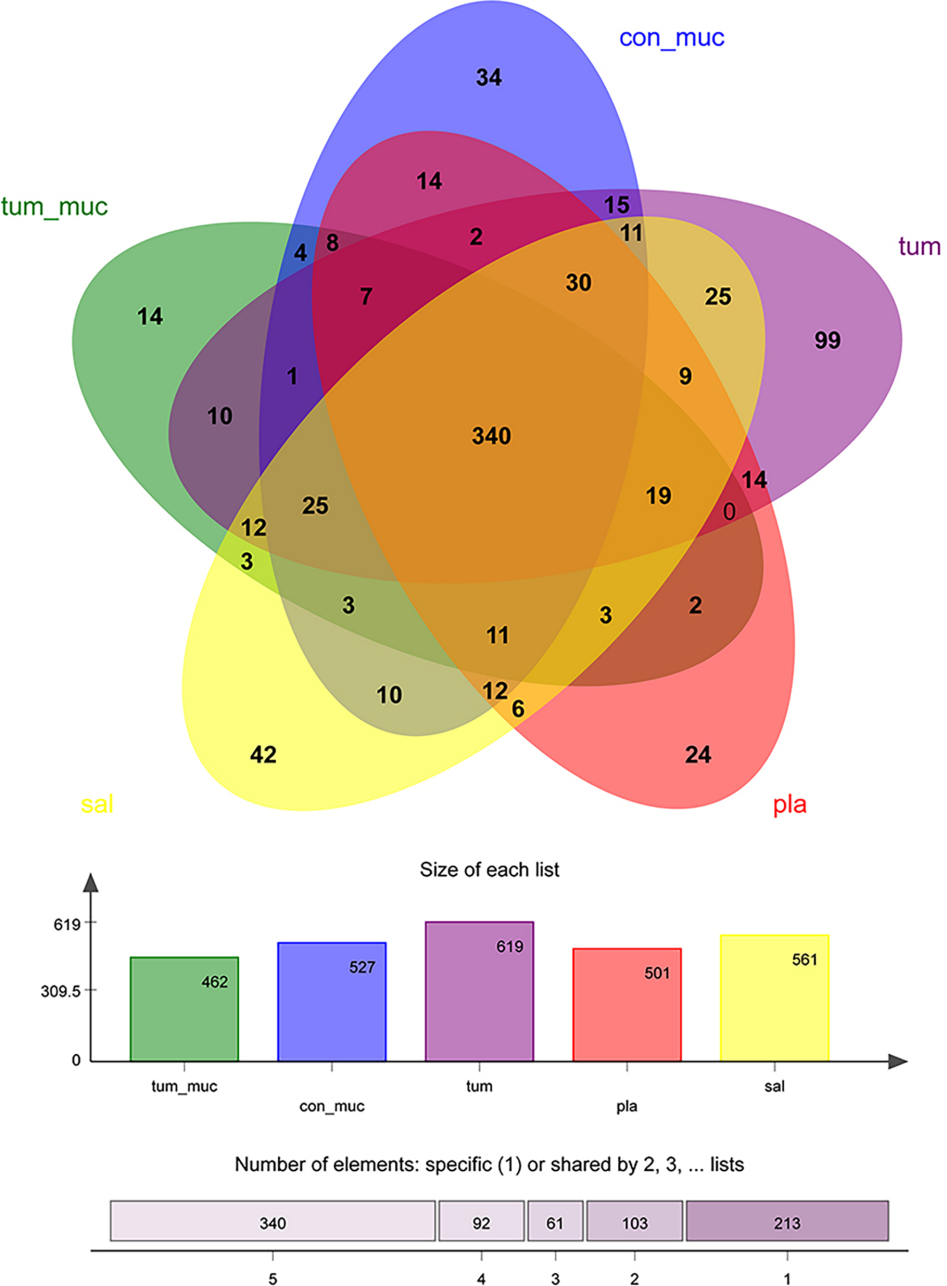
Venn diagram of the number of operational taxonomic unit (OTU) among normal mucosa, subgingival plaque, saliva, intratumoral tissue, and surface of tumor.

It can be seen from the Venn diagram (**Figure 6**) that the number of OTU that did not overlap on normal mucosa, subgingival plaque, saliva, intratumoral tissue, and surface of tumor was 34, 24, 42, 99, and 14, respectively, while the number of OTU that completely overlapped on the five sites was as high as 340, accounting for 55%~74% of the total number of each site. In other words, the composition of bacteria in saliva, subgingival plaque, surface of tumor, normal mucosa, and intratumoral tissue was very similar.

**Figure 7:**
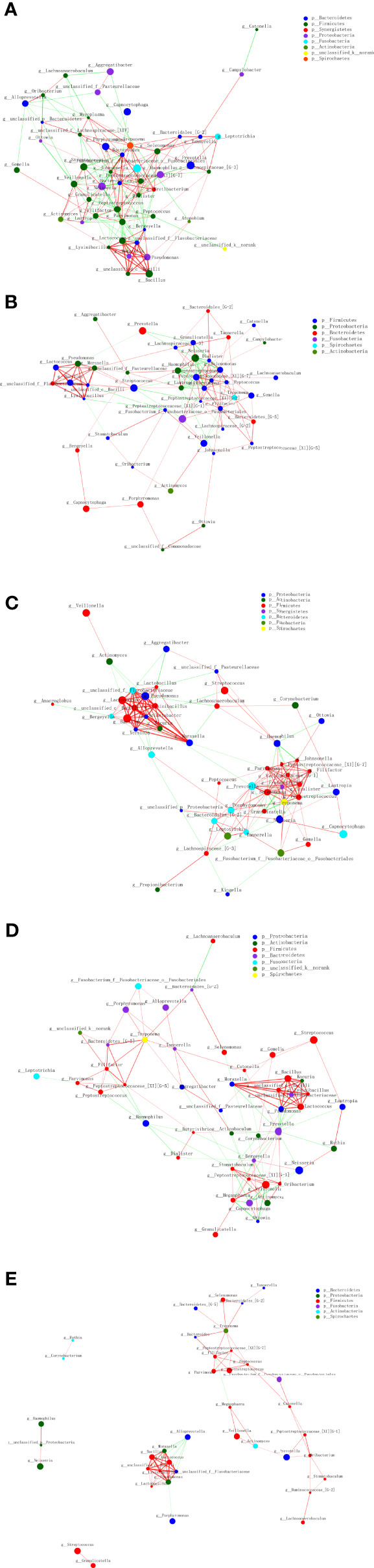
Interconnection of the oral squamous cell carcinoma (OSCC) and salivary and other sites bacteria. Tumor tissue **(A)**, the tumor surface **(B)**, subgingival plaque **(C)**, healthy mucosa **(D)**, and saliva **(E)**.

The size of nodes in **Figure 7** represents the abundance of genus, and different colors represent different genera. The colors of the lines indicate positive and negative correlations, red indicates positive correlation, and green indicates negative correlation. The thickness of the line indicates the correlation coefficient. The thicker the line, the higher the correlation between genera. The more lines, the more close the correlation.

It showed that the tumor site has the highest correlation between genera, and saliva site genus correlation is the lowest. In the tumor tissue, *Dialister*, *Johnsonella*, *Peptostreptococcus*, *Parvimonas*, and other bacteria were closely related to other bacteria. On the tumor surface, *Peptostreptococcus*, *Filifactor*, *Selenomonas*, and other bacteria were closely related to other bacteria. In the subgingival plaque, *Selenomonas*, *Peptostreptococcus*, *Prevotella*, and other bacteria were closely related to other bacteria, and the correlation was mostly positive. On the healthy mucosa, *Prevotella* was negatively associated with most microbes. In saliva, however, most of the microbes had a low microbiological correlation.

### Evaluation of Oral Squamous Cell Carcinoma Prediction Random Forest Models Based on Saliva Microbiome

Given the noninvasive collection process of salivary samples and the high sensitivity in OSCC sample identification, using the microbiome in salivary samples to identify potential OSCC patients would be a more appropriate choice of screening the OSCC patients. An additional random forest model was then built only using the 93 salivary samples, in which 47 were from the OSCC patients while the remaining 46 were from healthy controls (**Table 3**). The result showed that the model’s accuracy was 95.70%, and its sensitivity was 100%; i.e., four samples from healthy controls were misclassified as from OSCC patients. In addition to the model using only salivary samples, we wondered whether using samples of other sites could lead to the same performance. And the result is in the **Supplementary Material**.

**Table 3:** Prediction and observation of OSCC in saliva samples.

Observed	Predicted		Total
	Healthy controls	OSCC patients	
**Healthy controls**	42	4	46
**OSCC patients**	0	47	47

OSCC, oral squamous cell carcinoma.

Overfitting is a common concern in that a prediction model with high internal performance does not work well in other populations, especially in machine learning models. However, to evaluate such uncertainty, we carried out a batch of cross-validations. For each model, a training set containing 80% randomly selected samples was used to build a random forest model, and the rest of the samples were not used to build the model as the external test sets. Then, the average external accuracy of all the random forests provides an estimation of the model applied in external populations.

For the model built with OTUs in salivary samples, cross-validation showed an estimated external accuracy of 93.58%; i.e., 97 out of 1512 external test samples were misclassified in 84 external test sets containing 18 samples each. Still, no OSCC would be missed using the oral microbiome in salivary samples (**Table 4**).

**Table 4:** Prediction and observation of OSCC in saliva samples.

Observed	Predicted		Total
	Healthy controls	OSCC patients	(n = 1,512)
**Healthy controls**	659	97	756
**OSCC patients**	0	756	756

OSCC, oral squamous cell carcinoma.

The cross-validations of OSCC sample prediction random forests, i.e., using only salivary samples, with all the samples collected in five sites, suggested that the distinguished microbiome pattern in samples from OSCC individuals can also be used in external populations. Also, the sensitivity of external test samples still at 100% proves its high capacity in screening OSCC patients. Considering the noninvasive collection model, using random forests based on the microbiome in salivary samples would be strongly recommended to test whether the individuals are possible OSCC patients.

Another common concern is that given the well-known significant dependence on sample size, how many samples are required to build a model with favorable performance? Another batch of cross-validations, each with different sizes of training sets and test sets, was carried out to evaluate the dependence on sample size in OSCC identification using oral microbiome in salivary samples.

The sample sizes of training sets were set as 60% (56), 70% (65), 80% (75), and 90% (83) of all the 93 salivary samples. For each sample size, the random forest model was tested different times of a test sample size of 10,000 to obtain accuracy with comparable variations. The average accuracies of the tests with different sample sizes provided the association of the training sample sizes and the performance of OSCC identification models using the salivary microbiome.

**Figure 8:**
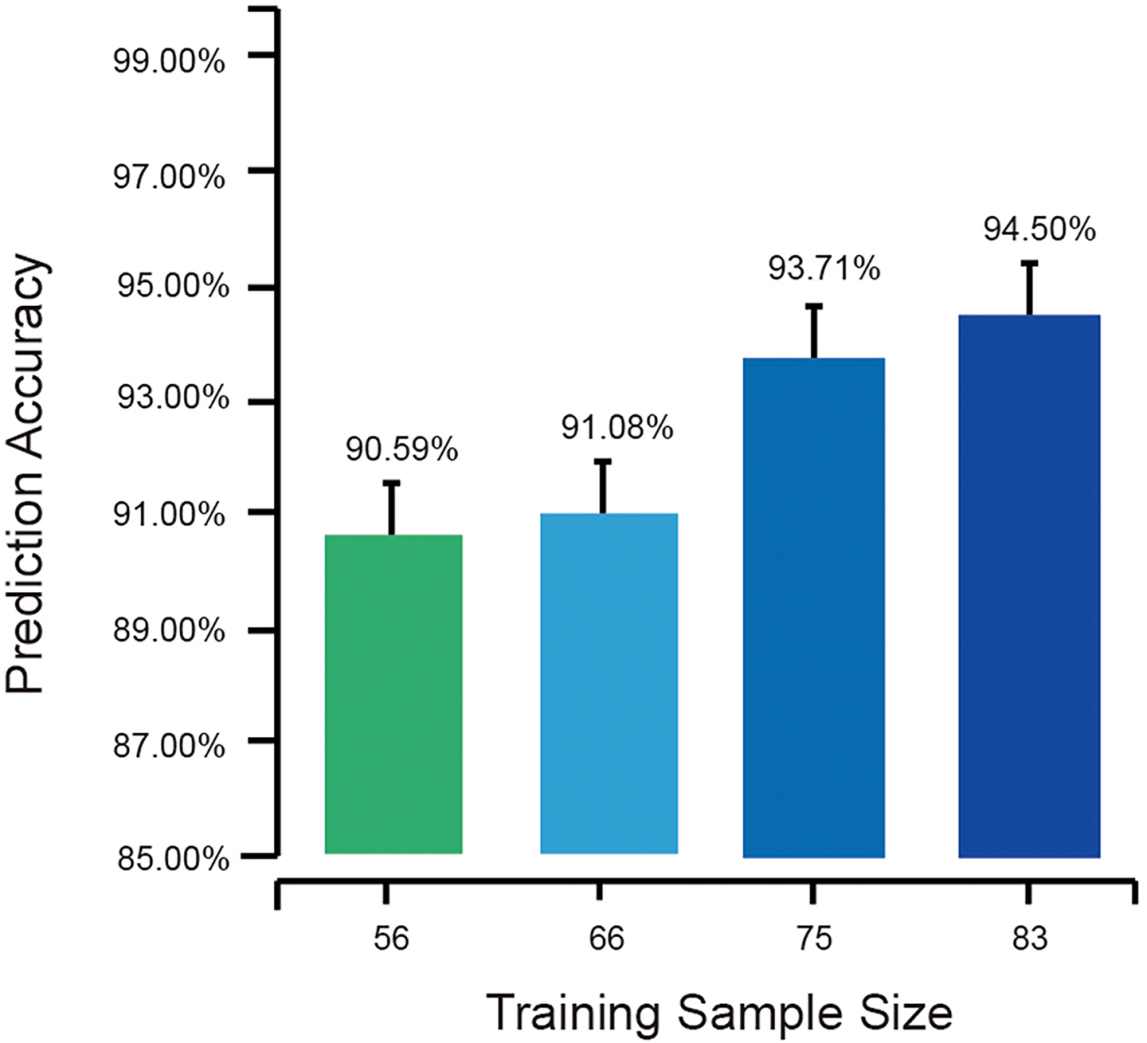
The average external prediction accuracies of random forests with different training sample sizes. The bottom part of the figure is truncated to present the differences in the error bar.

Cross-validations showed that as the training sample size increased, the random forests based on salivary samples became more accurate. Seventy-five samples could supply significantly high performance, while more training samples could give rise to even greater accuracy; i.e., with all the 93 samples in training set at 95.70% accuracy, another 1.20% improvement could be obtained over 94.50% with 83 samples in the training set (**Figure 8**). Also, the larger training sample size decreased the variance of the prediction accuracy, suggesting that higher accuracy can be obtained by a model with a large training set.

Further insight into >40,000 prediction results provided a good sample of this point. In the cross-validations, all the random forests with training sample sizes >80% (75) exhibited sensitivities of 100%; therefore, no OSCC would be misclassified. However, as the training sample size decreased, false-negative predictions were found, i.e., 15 patients in 5,130 OSCC patients with a training sample size of 56 and three patients in 4,998 OSCC patients with a training sample size of 65. An interesting finding is that all the 18 false-negative predictions happened in one same sample. This sample was found from an OSCC patient having early invasive carcinoma on the oral cavity floor. At the early stages of cancer development, the characteristics of the oral microbiome were close to those of healthy individuals compared with other OSCC patients. Such samples from early cancer development stages might be misclassified as from healthy individuals due to the partly changed microbial profiles, which would be identified correctly by the models with larger training sets.

This suggested the strategy of screening model development; the prediction random forest can first be built based on small sample sizes, such as those >50, and then the accuracy can be improved as the new samples are added to the training set to renew the basic model.

Also, the difference between groups of healthy individuals from different centers suggested that a continuous and dynamic renewal of the prediction model using new samples would be of necessity for a potential change in population applied. It is recommended for each center to address the differences between microbial profiles in different populations when building its own prediction random forest.

## Discussion

The human microbiome, a dynamic, interconnected ecosystem reflecting the locating environments, plays a central role in the process of development, health, and disease (Bracci, 2017; Cong and Zhang, 2018; Verma et al., 2018). Although the differences between microbiome in groups having different disorder statues might provide potential biomarkers, traditional analyses have low test power in identifying such differences due to the adverse effect of dimensionality. Although there is a deluge of data on the human microbiome, converting them into clinically meaningful insights remains challenging (Quince et al., 2009; Hu et al., 2013). Machine learning methods constitute proper tools for analyzing such high-dimensional datasets with a small sample size. For instance, Teng et al. (2015) developed a predictive model for early childhood caries (ECC) using oral microbiota by random forests machine learning algorithm innovatively, which became an asset for clinical work. The algorithm was also used in this study, indicating that OSCC can be diagnosed based on oral microbiota. Moreover, microbiota on any one of the five sites were useful for the diagnosis of OSCC. Thus, oral microbiota on any one of the five sites could be collected to diagnose OSCC in clinical practice.

Salivary samples would be an optimal choice for the OSCC preliminary diagnosis due to their advantages in the sample collection process. Early diagnosis plays a critical role in the treatment of OSCC, and many methods have been used in the diagnosis of OSCC. Compared with the traditional methods (CT, MRI, and PET), our novel model, based on oral microbiota, exhibited apparent advantages. First, compared with CT and PET, no radiation is involved during sample collection and examination. Second, the cost of 16S rRNA gene sequences is 20%–50% of CT/MRI for every patient and <20% of PET. Third, the examination is more convenient for patients than CT, MRI, and PET. This method only requires the collection of saliva and sequencing, without the need for professionals to purchase or learn other examination equipment, and can be easily implemented in oral medical clinics or hospitals. Fourth, the diagnosis of histopathological analysis usually takes about 3–5 days, because the preparation of tissue samples and the interpretation by the diagnostic physician are quite complex and rigorous. This method only requires sequencing and machine data processing, which will provide quick help for diagnosis. Besides, some studies have indicated that oral microbiota could provide a potential risk assessment for several other diseases, like dental caries (Stuckensen et al., 2000; Ng et al., 2005; Liao et al., 2011). Our investigations explored a novel method to detect OSCC at an early stage, expanding the application of oral microbiota in diagnosing oral diseases. Therefore, in the future, the analysis of oral microbiota might be included in annual physical examinations for large populations to detect the risk of different diseases. The selected people with a high risk of specific diseases could be referred to specialists for further confirmatory diagnosis. On the one hand, patients could benefit from the early diagnosis of the diseases; on the other hand, it could help reduce the social and public health expenses.

In the study, the accuracy of the diagnostic model was more favorable than that of the traditional methods. The accuracy of CT/MRI ranges from 66% to 86.4%. In recent years, ^18^F-FDG PET has been recommended in the diagnosis of OSCC patients (Kitajima et al., 2015). The accuracy of ^18^F-FDG PET ranges from 66.8% to 89.4%. In the present study, the accuracy of the novel model was 95%. Interestingly, there was no false-negative result in our diagnostic model. But there are still some false-positive individuals, and further confirmatory diagnosis could help exclude such cases.

In recent years, some studies indicated that oral microbial composition differed significantly from a healthy state to OSCC patients and non-tumoral to tumoral sites (Ahn et al., 2012; Pushalkar et al., 2012; Schmidt et al., 2014). Therefore, researchers tried to isolate some particular species and show their relationship with OSCC. In the present study, the results also provided evidence for some oral bacteria as potential research objects. As shown in **Table S2**, the top 10 features of oral microbiome in random forests were consistent with previously reported studies in which close relationships were detected between OSCC and the following bacterial species: *Porphyromonas*, *Fusobacterium*, *Prevotella*, *Leptotrichia*, *Moraxella*, *Bacillus*, and *Actinobacteria* (Sato et al., 2010; Al-Hebshi et al., 2015). Particularly, as pathogenic bacteria of periodontal disease, *P. gingivalis* and *Fusobacterium nucleatum* could promote oral carcinogenesis (Groeger et al., 2011; Gallimidi et al., 2015; Ha et al., 2015). *P. gingivalis* could promote immunoevasion of oral cancer by protecting cancer from macrophage attack and could facilitate cell migration, which was slightly enhanced by co-infection with *F. nucleatum* (Liu et al., 2020). *Prevotella* was found to have a close relationship with digestive tract cancers (Yang et al., 2009). Although other bacteria in the present study lacked in mechanism evidence, they provided clues for future studies to reveal the relationship between microorganisms and oral cancer.

In the present study, in one sample, the oral microbiome’s characteristics were close to those of healthy individuals. Further analysis indicated that it might be because the sample was collected from a patient in the early stages of OSCC, confirming previous research (Chocolatewala et al., 2010; Perera et al., 2016; Mukherjee et al., 2017), in which the microbiome changes continued with cancer development. The microbiome will change with the pathological environment during carcinogenesis.

In conclusion, using random forests and cross-validations, this study provided a method to build a diagnostic model based on oral microbiota, which could be applied to the diagnosis of OSCC in large populations accurately and conveniently without radiation before invasive procedures. Furthermore, this study provided an application sample to develop diagnostic models as an auxiliary diagnostic tool not only for OSCC but also for various tumors.

## Data Availability Statement

The datasets presented in this study can be found in online repositories. The names of the repository/repositories and accession number(s) can be found below: https://www.ncbi.nlm.nih.gov/, SRP119028.

## Ethics Statement

The institutional review board of the West China Hospital Stomatology of Sichuan University approved the study (Approval number: WCHSIRB-D-2013-047). The patients/participants provided their written informed consent to participate in this study.

## Author Contributions

XXZ: validation, methodology, formal analysis, data curation, writing—original draft, and writing—review and editing. YH: validation, methodology, data curation, writing—original draft, and writing—review and editing. XP: methodology, data curation, and writing—original draft. BL: methodology, data curation, and writing—original draft. QH: methodology and data curation. BR: supervision. ML: supervision. LL: resources. YL: supervision. GC: formal analysis and supervision. JL: funding acquisition and supervision. YM: conceptualization, methodology, formal analysis, writing—review and editing, and funding acquisition. XDZ: conceptualization, methodology, and funding acquisition. LC: conceptualization, methodology, writing—review and editing, and funding acquisition. All authors contributed to the article and approved the submitted version.

## Funding

This study was supported by the National Natural Science Foundation of China, 81870759 and 82071106 (LC), 81803332 (YM), and 81991500 and 81991501 (JL); the Sichuan Science & Technology Program 2018SZ0284 (YM); Innovative Research Team Program of Sichuan Province (LC); and the Chengdu Science & Technology Bureau 2018-YF05-01265-SN (YM).

## Conflict of Interest

The authors declare that the research was conducted in the absence of any commercial or financial relationships that could be construed as a potential conflict of interest.

## Publisher’s Note

All claims expressed in this article are solely those of the authors and do not necessarily represent those of their affiliated organizations, or those of the publisher, the editors and the reviewers. Any product that may be evaluated in this article, or claim that may be made by its manufacturer, is not guaranteed or endorsed by the publisher.
