# Supplementary material for: The Clinical Potential of Oral Microbiota as a Screening Tool for Oral Squamous Cell Carcinomas
Source: Front Cell Infect Microbiol. 2021 Aug 18;11:728933. doi: 10.3389/fcimb.2021.728933 (PMC8416267; doi:10.3389/fcimb.2021.728933)
Supplement: Supplementary file 1 [file DataSheet_1.docx]

Supplementary Material

# Random Forests Prediction Model of OSCC using microbiome profiles in samples collected in different sites

The mean size of each OTU, tumor, and the individual from which the sample was collected were used to build the random forests model to identify the samples from OSSC patients. The prediction result showed that the model had significant discrimination with 98.17% accuracy, i.e., only three samples out of 46 healthy controls were misclassified as from OSCC patients in all the 164 samples (Table S1).

This result suggested that the oral microbiome had a distinguished pattern in OSCC patients. Also, it is notable that only samples from healthy individuals were mistakenly predicted. Since no OSCC patients would be missed, this oral microbiome-based prediction model can serve as an early screening tool for OSCC to reduce unnecessary invasive histopathologic examinations.

The importance of OTUs in the random forests used microbiome in all the 164 samples of five sites and only 93 salivary samples were extracted. Ninety-four OTUs with a relative importance of >0.0001 in both random forests based on 93 salivary samples and all the 164 samples were illustrated in Figure S1. Although the relative importance of OTUs differed in microbiomes from salivary samples and all the samples, the whole pattern of important OTU seems similar in different samples.

Top 10 OTUs of each forest are presented in Table S2, in which seven OTUs were the same. Also, 25 (50%) and 76 (76%) in the top 50 and 100 significant OTUs were the same in both the 93 salivary samples and all the 164 samples from 5 sites. The detailed results are presented in Table S2. This suggested that the distinguished pattern of microbiomes existed in microbiomes from different oral sites, demonstrating the capacity of the salivary microbiome in identifying OSCC patients. These bacteria included Fusobacterium, Prevotella, Capnocytophaga, Prevotella, Moraxella, Peptococcus, Bacillus, Lactococcus, and so on.

For the model built with all the samples, cross-validation showed an estimated external accuracy of 96.67%, i.e., 55 out of 1650 external test samples were misclassified in 50 external test sets containing 33 samples each. Still, no OSCC would be misclassified using the oral microbiome in samples from all the five sites (Table S3).

# Supplementary Figures and Tables


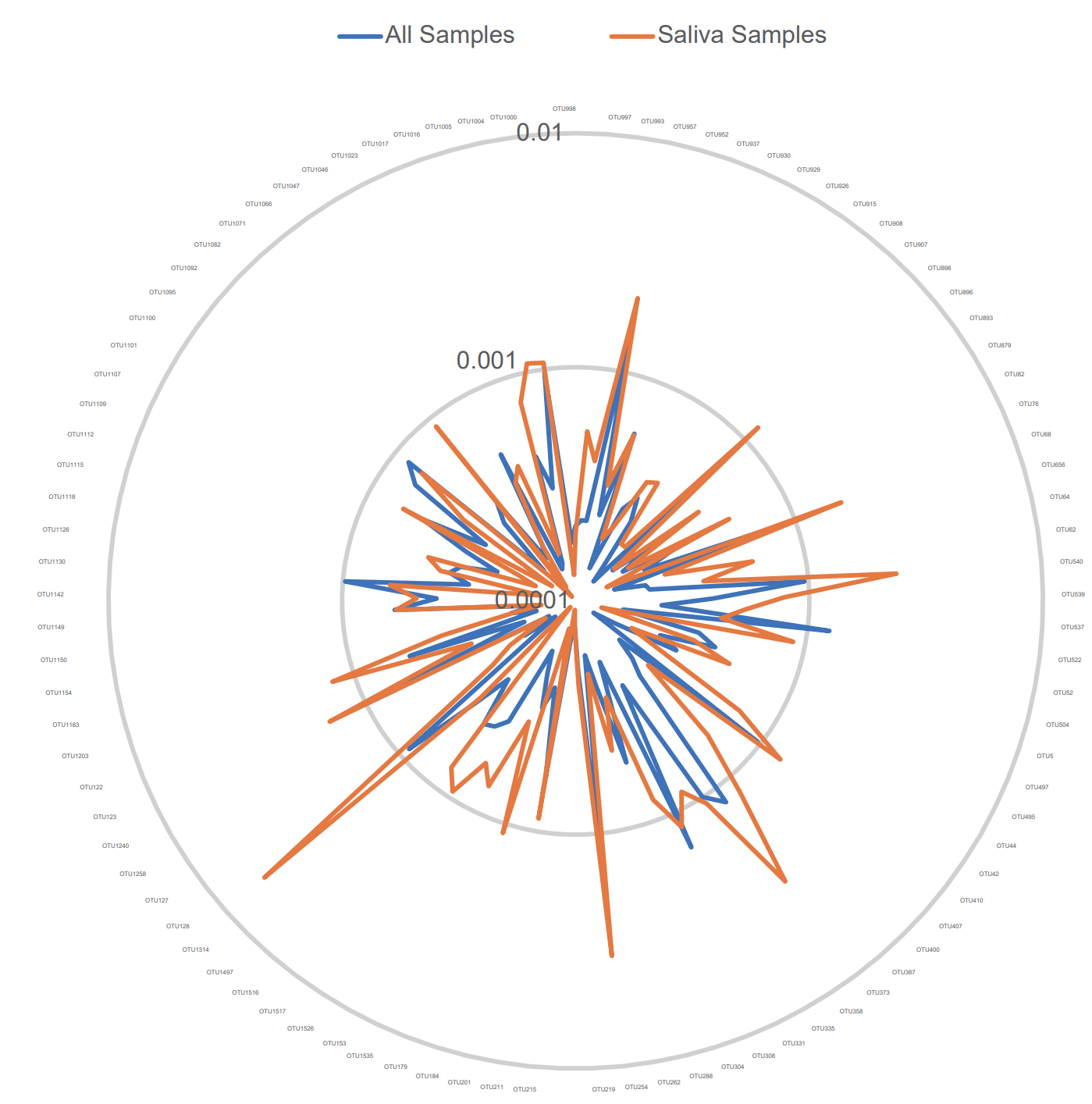


**Figure S1. Radar plot of 94 OTUs with a relative importance of >0.0001 in both random forests based on 93 salivary samples or all the 164 samples.**

**
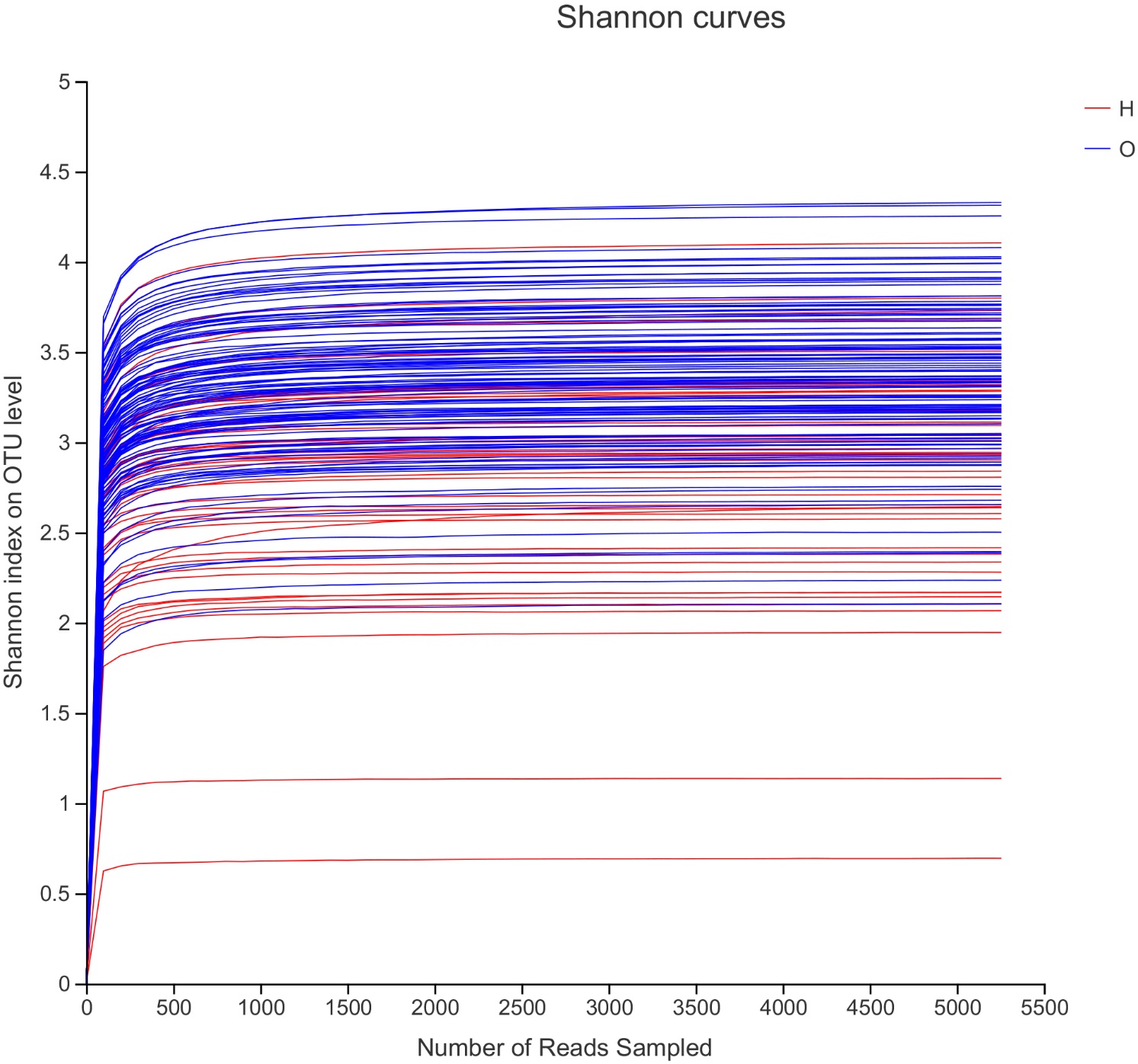
**

**Figure S2. Rarefaction analysis of bacterial 16S rRNA gene sequences in OSCC group and healthy group.**

**Table S1. Samples in different groups.**

| **Observed** | **Predicted** | | **Total** |
| --- | --- | --- | --- |
|  | Healthy Controls | OSCC Patients |  |
| **Healthy controls** | 43 | 3 | 46 |
| **OSCC Patients** | 0 | 118 | 118 |

Table S2. Top 10 features in random forests based on samples from different sites.

| **Saliva** | **T** | **OTU186** | **OTU128** | **OTU254** | **OTU187** | **OTU1522** | **OTU373** | **OTU291** | **OTU540** | **OTU480** |
| --- | --- | --- | --- | --- | --- | --- | --- | --- | --- | --- |
| **Genus** |  | Fusobacterium | Prevotella | Capnocytophaga | Unclassified (Bacilli) | Unclassified (Actinobacteria) | Prevotella | Moraxella | Peptococcus | Bacillus |
| **Importance** | 46 | 6.09 | 6.06 | 3.36 | 3.22 | 3.17 | 3.15 | 2.63 | 2.38 | 2.15 |
| **(10^-3^)** |  |  |  |  |  |  |  |  |  |  |
| **Rank in Saliva** | 1 | 2 | 3 | 4 | 5 | 6 | 7 | 8 | 9 | 10 |
| **All Sites** | **T** | **OTU1522** | **OTU186** | **OTU480** | **OTU432** | **OTU187** | **OTU291** | **OTU533** | **OTU419** | **OTU254** |
| **Genus** |  | Unclassified (Actinobacteria) | Fusobacterium | Bacillus | Bacillus | Unclassified (Bacilli) | Moraxella | Lactococcus | Bacillus | Capnocytophaga |
| **Importance** | 31.69 | 4.12 | 3.44 | 3.24 | 2.66 | 2.12 | 2.06 | 1.95 | 1.86 | 1.68 |
| **(10^-3^)** |  |  |  |  |  |  |  |  |  |  |
| **Rank in all Sites** | 1 | 2 | 3 | 4 | 5 | 6 | 7 | 8 | 9 | 10 |

Table S3. Prediction and observation of OSCC in samples from all 5 sites.

| **Observed** | **Predicted** | | **Total** |
| --- | --- | --- | --- |
|  | Healthy Controls | OSCC Patients | **(n=1650)** |
| **Healthy Controls** | 403 | 55 | 458 |
| **OSCC Patients** | 0 | 1192 | 1192 |

Table S4. Table for Read count statistics of all samples.

| **Sample\Info** | **Read count** |
| --- | --- |
| **1** | 36021 |
| **2** | 32961 |
| **90448** | 46501 |
| **101809** | 33365 |
| **101825** | 37965 |
| **101858** | 39444 |
| **101859** | 39655 |
| **101870** | 44892 |
| **101875** | 47730 |
| **101880** | 40971 |
| **101909** | 35553 |
| **101921** | 36730 |
| **101924** | 49961 |
| **101926** | 50680 |
| **102001** | 28599 |
| **102008** | 40343 |
| **102024** | 48275 |
| **102042** | 48282 |
| **102043** | 52175 |
| **102626** | 42679 |
| **102637** | 48657 |
| **102699** | 66772 |
| **10a** | 17055 |
| **10b** | 23808 |
| **10c** | 20290 |
| **10d** | 29517 |
| **11a** | 16748 |
| **11b** | 23935 |
| **11c** | 29515 |
| **11d** | 21361 |
| **12b** | 23045 |
| **12c** | 24881 |
| **12d** | 24843 |
| **13a** | 17669 |
| **13b** | 20042 |
| **13c** | 20693 |
| **13d** | 25017 |
| **14a** | 17382 |
| **14c** | 23094 |
| **14d** | 23624 |
| **14e** | 18409 |
| **15b** | 18638 |
| **15d** | 23481 |
| **16a** | 19116 |
| **16b** | 21792 |
| **16c** | 21114 |
| **16d** | 25792 |
| **16e** | 22437 |
| **17a** | 21047 |
| **17c** | 20765 |
| **17d** | 23653 |
| **17e** | 18525 |
| **18a** | 18697 |
| **18b** | 19998 |
| **18c** | 20572 |
| **18d** | 22791 |
| **18e** | 20293 |
| **19a** | 18455 |
| **19b** | 19142 |
| **19c** | 34055 |
| **19d** | 27455 |
| **19e** | 19284 |
| **1a** | 17933 |
| **1b** | 16247 |
| **1e** | 23946 |
| **20a** | 17119 |
| **20b** | 20073 |
| **20c** | 22813 |
| **20d** | 27766 |
| **20e** | 22819 |
| **21a** | 14583 |
| **21c** | 23886 |
| **21d** | 31132 |
| **21e** | 19415 |
| **22a** | 19642 |
| **22b** | 20440 |
| **22d** | 28650 |
| **22e** | 21749 |
| **23c** | 24190 |
| **23d** | 23833 |
| **23e** | 16019 |
| **24b** | 21285 |
| **24c** | 26303 |
| **24d** | 20249 |
| **24e** | 20829 |
| **25a** | 20025 |
| **25c** | 20839 |
| **25d** | 26801 |
| **2c** | 20556 |
| **2d** | 30574 |
| **2e** | 27082 |
| **3a** | 18249 |
| **3b** | 17534 |
| **3c** | 17447 |
| **3d** | 21648 |
| **4a** | 21870 |
| **4c** | 20060 |
| **4d** | 22106 |
| **5a** | 19577 |
| **5c** | 23293 |
| **5d** | 22425 |
| **5e** | 25927 |
| **60KQ** | 46151 |
| **63KQ** | 44902 |
| **64KQ** | 47549 |
| **65KQ** | 46111 |
| **66KQ** | 33831 |
| **67KQ** | 45534 |
| **69KQ** | 44292 |
| **6b** | 22102 |
| **6c** | 35553 |
| **6d** | 23716 |
| **6e** | 26079 |
| **70KQ** | 43936 |
| **71KQ** | 41161 |
| **72KQ** | 39973 |
| **73KQ** | 45737 |
| **7b** | 26089 |
| **7d** | 20649 |
| **8a** | 17021 |
| **8b** | 22739 |
| **8c** | 22025 |
| **8d** | 22510 |
| **8e** | 20380 |
| **9a** | 20912 |
| **9b** | 25664 |
| **9c** | 17264 |
| **9d** | 22877 |
| **9e** | 22805 |
| **CYY** | 35143 |
| **ES003** | 42871 |
| **HN** | 31880 |
| **QY** | 33352 |
| **SRR6185527** | 119585 |
| **SRR6185532** | 76260 |
| **SRR6185533** | 81204 |
| **SRR6185600** | 66390 |
| **SRR6185601** | 30345 |
| **SRR6185608** | 119181 |
| **SRR6185609** | 25326 |
| **SRR6185610** | 104018 |
| **SRR6185611** | 83335 |
| **SRR6185612** | 27356 |
| **SRR6185613** | 84961 |
| **SRR6185614** | 32572 |
| **SRR6185615** | 59165 |
| **SRR6185616** | 28813 |
| **SRR6185617** | 72424 |
| **SRR6185618** | 49085 |
| **SRR6185619** | 25654 |
| **SRR6185620** | 80156 |
| **SRR6185621** | 69902 |
| **SRR6185622** | 51911 |
| **SRR6185623** | 85782 |
| **SRR6185624** | 53947 |
| **SRR6185625** | 100960 |
| **SRR6185626** | 75326 |
| **SRR6185627** | 50125 |
| **WHLT** | 39469 |
| **WHYT** | 95376 |
| **WZ** | 30685 |
| **YJZ** | 32642 |
| **YS003** | 49238 |
| **ZCG** | 34565 |

# SI References

Xun, Z., Q. Zhang, T. Xu, N. Chen & F. Chen (2018) Dysbiosis and Ecotypes of the Salivary Microbiome Associated With Inflammatory Bowel Diseases and the Assistance in Diagnosis of Diseases Using Oral Bacterial Profiles. Frontiers in Microbiology, 9, 17.doi:10.3389/fmicb.2018.01136
